# Flexible thin-film acoustic wave devices with off-axis bending characteristics for multisensing applications

**DOI:** 10.1038/s41378-021-00325-3

**Published:** 2021-11-26

**Authors:** Zhangbin Ji, Jian Zhou, Huamao Lin, Jianhui Wu, Dinghong Zhang, Sean Garner, Alex Gu, Shurong Dong, YongQing Fu, Huigao Duan

**Affiliations:** 1 College of Mechanical and Vehicle Engineering, Hunan University, 410082 Changsha, China; 2 Shanghai Industrial μTechnology Research Institute (SITRI), 235 Chengbei Rd, 201800 Shanghai, China; 3 Corning Research & Development Corporation, One River Front Plaza, Newark, NY 14831 USA; 4 College of Information Science and Electronic Engineering, Zhejiang University, 310027 Hangzhou, China; 5 Faculty of Engineering and Environment, Northumbria University, Newcastle upon Tyne, NE1 8ST UK

**Keywords:** Electrical and electronic engineering, Physics

## Abstract

Flexible surface acoustic wave (SAW) devices have recently attracted tremendous attention for their widespread application in sensing and microfluidics. However, for these applications, SAW devices often need to be bent into off-axis deformations between the acoustic wave propagation direction and bending direction. Currently, there are few studies on this topic, and the bending mechanisms during off-axis bending deformations have remained unexplored for multisensing applications. Herein, we fabricated aluminum nitride (AlN) flexible SAW devices by using high-quality AlN films deposited on flexible glass substrates and systematically investigated their complex deformation behaviors. A theoretical model was first developed using coupling wave equations and the boundary condition method to analyze the characteristics of the device with bending and off-axis deformation under elastic strains. The relationships between the frequency shifts of the SAW device and the bending strain and off-axis angle were obtained, and the results were identical to those from the theoretical calculations. Finally, we performed proof-of-concept demonstrations of its multisensing potential by monitoring human wrist movements at various off-axis angles and detecting UV light intensities on a curved surface, thus paving the way for the application of versatile flexible electronics.

## Introduction

Acoustic wave devices (especially those based on surface acoustic waves, SAWs) are attracting substantial interest as sensing platforms to detect physical, chemical and biological substances^1–3^. They offer merits such as high sensitivity, a low detection limit (as the energy of the waves is mostly concentrated on the surface) and wireless-passive detection. They are also highly desirable for emerging technologies and integrated devices, including acoustofluidics^4,5^, lab-on-chip applications^6^, next-generation quantum communications^7^, and integrated microwave-photonics signal processing^8^. Conventional SAW devices are fabricated on single-crystal piezoelectric substrates, making them rigid and unsuitable for application in flexible electronics. This severely restricts their application on the bent or curved surfaces of next-generation prosthetics, soft robotics, personalized healthcare systems and flexible printed circuits. This engineering challenge has motivated research on new materials, novel device architectures, and advanced manufacturing technologies.

Recently, to achieve mechanically robust, flexible and adaptive sensing microelectronics, flexible SAW devices with good flexibility and foldability have been developed. In 2013, Jin et al. fabricated flexible SAW sensing devices by depositing ZnO piezoelectric thin films on low-cost and commercially available polyimide (PI), demonstrating their excellent temperature sensing capability^9^. After that, various and flexible SAW applications, including strain sensors^10,11^, humidity sensors and respiration monitoring^12^, UV sensors^13^, and microfluidics^14^, have been reported using polymer substrates or metallic foil/sheet substrates. However, due to the significant dissipation of sound waves and energy, low temperature tolerance and poor adhesion of thin films on polymer substrates, it is a very large challenge to use these polymer-based thin-film SAW devices for wide-range environmental sensing (for example, a high-temperature environment) or high-performance microfluidics and lab-on-chip applications. On the other hand, metallic foils such as Al foils easily undergo plastic deformation and show poor flexibility; additionally, they do not easily recover to their original states when they are bent. In addition, both polymer- and metallic foil-based SAW devices have difficulties in wafer level production, and they are incompatible with the IC process.

To address these limitations, Luo’s group^15^ and our group^16,17^ reported flexible SAW devices based on ZnO films on flexible glass. Ultrathin flexible glass is a suitable inorganic substrate material for flexible SAW devices because it demonstrates good recovery after being bent, low acoustic energy dissipation, excellent thermal and environmental stability, and the capability of producing wafers (up to a 6-inch scale). However, ZnO-based SAW devices are not stable in extremely acidic and alkali environments along with high-temperature environments^18^, which inhibits their widespread application. In contrast, AlN films have better mechanical and chemical stability and higher wave propagation velocity and should be suitable candidates for high-performance sensors for applications in acidic/alkali and high-temperature environments^19^. However, AlN/glass flexible-based SAW devices have never been reported. In addition, there are few studies on the bending characteristics and mechanisms of flexible SAW devices under different bending strains and off-axis deformations. There is also an urgent need to differentiate the effects of bending strain and off-axis angle of strain (along the acoustic-wave propagation direction and/or bending direction) on the device’s sensing performance.

**Fig. 1:**
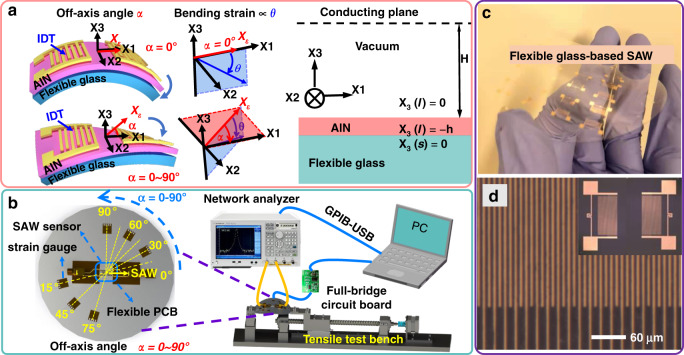
Model of bending and off-axis deformation and the strain testing system for flexible SAW devices. **a** Schematic diagrams of flexible and layered SAW devices at different off-axis angles α and bending strains (which is proportional to the bending angle θ); **b** schematic view of the strain testing system used for flexible SAWs at various off-axis angles α; **c** fabricated flexible SAW device on an ultrathin flexible glass substrate, showing its good flexibility and wafer-scale level; and **d** microscopic images of fabricated flexible SAW devices, showing the whole device and well-defined IDTs with a periodicity of 20 µm.

Herein, we fabricated AlN/glass flexible SAW devices by depositing high-quality AlN films onto flexible glass substrates, as illustrated in Fig. 1. A theoretical model was developed using the coupling wave equation and boundary condition method to systematically analyze the bending and off-axis deformation characteristics of flexible SAW devices under elastic strains (Fig. 1a). Variations in frequency shifts were obtained with different bending strains, IDT periods and off-axis angles between the acoustic-wave propagation direction and bending direction. The results showed that the experimental results are identical to those from the theoretical calculations. To demonstrate their practical application, AlN/glass flexible SAW devices were applied to monitor the bending of a human wrist. Furthermore, our flexible SAW sensors also demonstrated good capabilities for UV sensing on a curved substrate surface.

## Modeling the bending and off-axis deformation of flexible SAW devices

We first used coupling wave equations and boundary condition methods to systematically analyze the bending and off-axis deformation characteristics under elastic strains for flexible glass-based SAW devices.

The frequency of the flexible SAW device changes with its bending and off-axis deformation due to the strain-induced changes in both SAW velocity and wavelength (due to IDT deformation in the direction of SAW propagation). To investigate these in detail, the above two components are considered independently. The velocity changes under the applied stress can be calculated by a modified form of equations of motion that considers the perturbation of applied strains^20^. When an AlN/flexible glass layered device is under a bending strain, the coupling wave equations are^21^

1 $$\sigma _{jk}\frac{{\partial ^2u_i}}{{\partial x_j\partial x_k}} - \rho \frac{{\partial ^2u_i}}{{\partial t^2}} + C_{ijkl}\frac{{\partial ^2u_k}}{{\partial x_l\partial x_j}} + e_{jkl}\frac{{\partial ^2\phi }}{{\partial x_k\partial x_j}} = 0$$

2 $$e_{jkl}\frac{{\partial ^2u_k}}{{\partial x_l\partial x_j}} - \varepsilon _{jk}\frac{{\partial ^2\phi }}{{\partial x_k\partial x_j}} = 0$$

where i, j, k, l = 1, 2, 3, the Einstein summation convention is indicated by repeated indices; σ_jk_ is the initial stress; u is the mechanical displacement; ρ is the density; φ is the electrical potential; and C_ijkl_, e_jkl_, and ε_jk_ are elastic stiffness constants, piezoelectric constants and dielectric constants, respectively.

A solution for the displacement vector of the AlN/glass flexible SAW device is a linear combination of fundamental solutions in the form of ref. ^20^

3 $$u_k = e^{j\omega t}\beta _k\exp \left[ { - j\frac{w}{v}\left( {x_1 + \gamma x_3} \right)} \right]\left( {k = 1,2,3,4} \right)$$

where *γ* is a complex normalized transverse wave number and *v* is the acoustic-wave velocity. Here, u_4_ represents electrical potential *φ*.

By substituting Eq. (3) into Eqs. (1) and (2) and ignoring the effects of horizontal shear waves due to the type of crystal structure of AlN (with its dominant (0002) orientation) and flexible glass, we then obtain the simplified Christoffel equations for the AlN piezoelectric layer and flexible glass substrate, as shown in Eq. (4) and Eq. (5), respectively.

4 $$\left[ {\begin{array}{*{20}{c}} {\Gamma _{11} - \rho v^2} & {\Gamma _{13}} & {\Gamma _{14}} \\ {\Gamma _{13}} & {\Gamma _{33} - \rho v^2} & {\Gamma _{34}} \\ {\Gamma _{14}} & {\Gamma _{34}} & {\Gamma _{44}} \end{array}} \right]\left[ {\begin{array}{*{20}{c}} {\beta _{1\left( L \right)}} \\ {\beta _{3\left( L \right)}} \\ {\beta _{4\left( L \right)}} \end{array}} \right] = 0$$

5 $$\left[ {\begin{array}{*{20}{c}} {\Gamma _{11} - \rho v^2} & {\Gamma _{13}} \\ {\Gamma _{13}} & {\Gamma _{33} - \rho v^2} \end{array}} \right]\left[ {\begin{array}{*{20}{c}} {\beta _{1\left( S \right)}} \\ {\beta _{3\left( S \right)}} \end{array}} \right] = 0$$

The determinants of these five equations with three variables *β*_*1(L)*_, *β*_*2(L)*_, *β*_*4(L)*_ and another two variables *β*_*1(S)*_*,* *β*_*3(S)*_ must be zero for nontrivial solutions. We can then obtain a six-order polynomial equation and a four-order polynomial equation in *γ*. In the piezoelectric material layer, there are six different roots of $\gamma _L^{\left( q \right)}$, q = 1–6, and all six $\gamma _L^{\left( q \right)}$ are useful. In the substrate layer, there are four different roots of $\gamma _L^{\left( q \right)}$, q = 1–4, and two values of $\gamma _S^{\left( q \right)}$, for which Im $\gamma _S^{\left( q \right)}$ > 0 are allowed because of the attenuation of the acoustic wave in the substrate. For each$\gamma$, the relevant $\beta _k$can be obtained.

Based on these, the complete solutions for both the piezoelectric layer and substrate can be given by

6 $$u_k\left( L \right) = \mathop {\sum}\limits_{q = 1}^{Q\left( L \right)} {e^{j\omega t}B_{\left( L \right)}^{\left( q \right)}\beta _{k\left( L \right)}^{\left( q \right)}\exp \left[ { - j\frac{w}{v}\left( {x_1 + \gamma ^{\left( q \right)}x_3} \right)} \right],Q\left( L \right) = 6}$$

7 $$u_k\left( S \right) = \mathop {\sum}\limits_{q = 1}^{Q\left( S \right)} {e^{j\omega t}B_{\left( S \right)}^{\left( q \right)}\beta _{k\left( S \right)}^{\left( q \right)}\exp \left[ { - j\frac{w}{v}\left( {x_1 + \gamma ^{\left( q \right)}x_3} \right)} \right],Q\left( S \right) = 2}$$

To obtain the acoustic-wave velocity, four boundary conditions are given^20^ and shown in the SI materials. The four boundary conditions can form a set of 8 homogeneous equations (SI materials) for two variables $B_{\left( S \right)}^{\left( q \right)}$ and six variables $B_{\left( L \right)}^{\left( q \right)}$. The determinants of these equation sets should be zero for nontrivial solutions. Then, a transcendental equation for the determination of *v* is obtained. An iterative process was further used in the computation to find the value of v.

When the applied strain is a perturbation to the propagating SAW, the applied strain not only modifies the equation of motion but also changes the material constants. There are three independent perturbed material constants influencing the acoustic velocity under stress^21^: the initial stress (σ_ij_), material elastic constants (*C*_ijkl_), and material density (ρ). The original parameters were substituted with these changed parameters to calculate the velocity changes under different strains and applied off-axis angles.

A MATLAB program was developed to conduct the iteration calculation of SAW velocities as a function of applied strain. The strain sensitivity (S) is defined as

8 $$S = \frac{{\Delta f}}{{\Delta \varepsilon }} = \frac{{\left( {\Delta v/v - \Delta \lambda /\lambda } \right).f_0}}{{\Delta \varepsilon }}$$

where *f*_*0*_ is the resonant frequency under zero strain. All the results of the detailed theoretical calculation are provided in the SI materials.

### Experimental section

The flexible SAW devices were fabricated on flexible Corning Willow glass substrates (100 μm in thickness and 75 mm in diameter), as illustrated in Fig. 1(c). Piezoelectric AlN films were deposited onto flexible glass substrates using a reactive magnetron sputtering system with a pure Al target and a gas mixture of N_2_/Ar. The optimal deposition conditions were a substrate temperature of 200 °C, a deposition pressure of 0.43 Pa, and a N_2_/Ar gas ratio of 1:5. The pulsed DC power was 10 kW, and the RF bias power was 160 W. The crystal orientation of the AlN film was characterized using X-ray diffraction (XRD-6000) with a Cu-*Kα* radiation source and a scanning range of 2*θ* = 20°–70°. Crystallite sizes were calculated using the Debye–Scherrer formula based on the full width at half maximum (FWHM) of the AlN diffraction peak (*β* in radians): *D* = *Kλ/(βcosθ)*^16^, where *K* is the shape factor of the average crystallite with a value of 0.94, *λ* is the X-ray wavelength (1.5405 Å for a Cu target), *θ* is the Bragg angle, and *D* is the mean crystallite gain size normal to diffracting planes. The residual strain of the film was calculated from *ε*_*z*_ = *(c* − *c0)/c0*^16^, where *c*_0_ is the strain-free lattice constant and *c is* the lattice constant, which is equal to twice the interplanar spacing *d*, measured from the position of the (0002) peak using the Bragg equation. Scanning electron microscopy (SEM, Sigma-300, ZEISS, Germany), atomic force microscopy (AFM, Dimension Icon, Bruker) and transmission electron microscopy (TEM, JEM-2100Plus, JEOL, Japan) were applied to characterize the cross-sectional morphologies, surface topography and lattice structure of the AlN films.

Conventional photolithography and lift-off processes were used to fabricate AlN/glass-based flexible SAW devices with different wavelengths (λ) of 12, 16, 20, and 24 μm. The flexible SAW device had 50 pairs of IDTs with a metallization ratio of 0.5, reflectors of 100 pairs, an aperture length of 200 *λ*, and a center distance between the two ports of 150 *λ*. Figure 1(d) shows an optical image of the fabricated AlN/glass-based flexible SAW device, demonstrating its good flexibility and uniform interdigitated structure.

For the bending tests, the fabricated SAW devices were diced into small devices (1.5 × 1.5 cm) from the glass wafer, as shown in Fig. 1(b), and then bonded onto a 1 mm thick stainless steel plate using ethyl α-cyanoacrylate adhesive (No. 502 glue). They were then electrically connected to the flexible printed circuit board (PCB, which was mounted onto a steel plate) using conductive silver paste. A tensile tester was used to bend the steel plate and apply the bending strain to the flexible SAW device. To investigate the effect of the off-axis angle (e.g., the angle between the acoustic-wave propagation direction and the bending direction) on the frequency responses of flexible SAW devices, we set the off-axis angles at 0°, 15°, 30°, 45°, 60°, 75°, and 90° by rotating the stainless steel plate. Therefore, strain was applied to the surface acoustic-wave device in different off-axis directions. Seven standard strain gauges were glued near the SAW sensor, which provided the strain readings and calibrated the dynamic strains. The transmission spectra of the flexible SAW devices were obtained using a vector network analyzer (3656D, Ceyear, China), and a LabVIEW program was developed to automatically record the frequency changes as a function of time at different bending strains and off-axis angles.

## Results and discussion

**Fig. 2:**
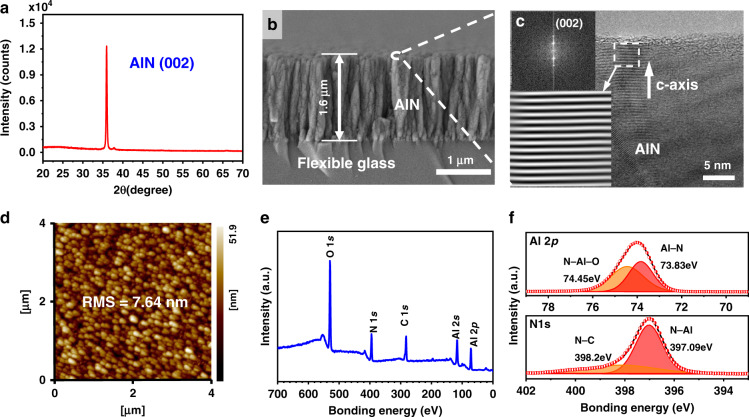
Characterization of AlN thin film deposited on the flexible glass substrate. **a** XRD pattern of the AlN film on flexible glass, showing strong (0002) orientation; **b** SEM image of the cross-section of the AlN film on flexible glass; **c** HRTEM image of AlN film, and the inset figure is FFT image; **d** AFM image of the AlN film on flexible glass, showing the smooth surface; **e** XPS survey spectrum of the AlN films on flexible glass; and **f** XPS high-resolution spectra of Al 2p and N 1 s of the AIN films.

Figure 2a shows the XRD pattern of the AlN film, which shows a single peak at an angle of ∼36°, corresponding to the AlN (0002) crystal orientation. The full width at half maximum (FWHM) is 0.281°, corresponding to the calculated mean grain size of approximately 29.7 nm. The axial stress of the film was estimated to be 42.25 MPa based on the lattice constant obtained through XRD patterns, showing that the deposited film has a low tensile residual stress on the flexible glass substrate. Figure 2b is a cross-section SEM image of the AlN film on flexible glass, which shows a vertically columnar structure. Figure 2c is a high-resolution TEM (HRTEM) image, and the corresponding fast Fourier transformation (FFT) image (inset) of the AlN film on flexible glass reveals that the AlN grains are well textured along the (0002) orientation. Figure 2d shows the surface morphology and roughness of the film obtained using AFM. The root mean square (RMS) roughness was measured to be 7.64 nm over an area of 4 × 4 μm^2^. The preferred *c*-axis orientation, low residual stress and small value of roughness of the AlN films are critical for high-performance flexible SAW devices.

Figure 2e shows a survey spectrum of the AlN films obtained using X-ray photoelectron microscopy (XPS), in which the photoelectron peaks of Al, N, C, and O can be detected. The N 1 s and Al 2p peaks in the XPS spectra confirm the formation of Al–N structures^22,23^. The binding states of these elements were further investigated using XPS, and the results are shown in Fig. 2f. Two binding-energy components of N 1 s were obtained after peak deconvolution using a curve-fitting program. The peak at a binding energy of 397.09 eV corresponds to aluminum–nitrogen (Al–N) bonds. This energy agrees well with the previously published values of AlN films^22^. For the binding-energy spectrum of Al 2p, two peaks at binding energies of 74.4 and 73.8 eV were deconvoluted for the best fitting of the spectrum. The lower binding-energy (73.8 eV) component can be attributed to aluminum in AlN^23^. The Al 2p peak, which appears at 74.4 eV, corresponds to aluminum in its oxide states^24^. The formation of a protective layer of aluminum oxides on the surface of AlN when exposed to the atmosphere has been well documented^25^.

Transmission (S_21_) spectra of the flexible SAW devices at different wavelengths are shown in Fig. S1. All these flexible SAW devices show well-defined Rayleigh resonant peaks. The resonant frequencies are 290.1, 211.8, 166.9, and 139 MHz for the SAW devices with wavelengths of 12, 16, 20, and 24 μm, respectively. The phase velocities (v_p_) of these SAW devices, V_p_ = *λ*f, were calculated to be 3481.50, 3388.32, 3340.40, and 3336.24 m·s^−1^, respectively, decreasing gradually with an increase in wavelength from 12 to 24 μm. When the wavelength is increased, more energy is dispersed into the flexible glass (3200 m·s^−1^), which has a lower wave propagation velocity than that of the AlN film (~5600 m·s^−1^), thus leading to a lower wave velocity in the layered structure. Discussions of device performance between our flexible AlN/glass SAW devices and the previous flexible SAW devices are shown in the SI materials.

### Bending and off-axis deformation characteristics of the flexible SAW device

**Fig. 3:**
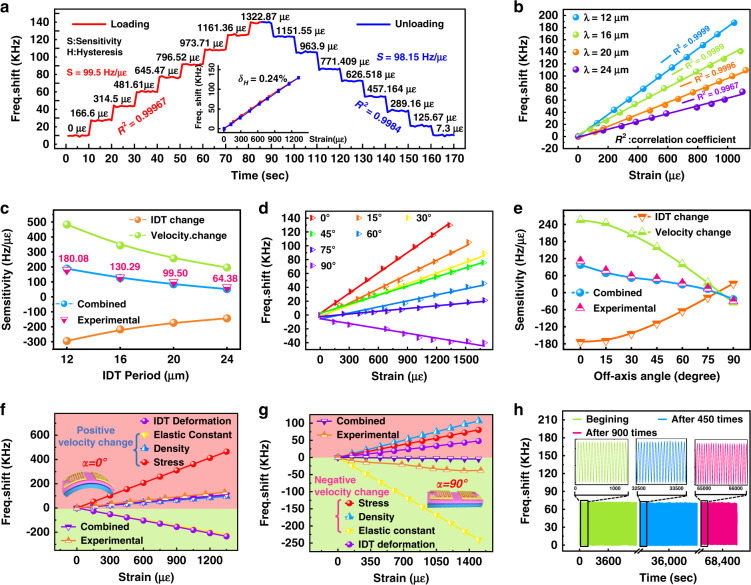
Bending and off-axis deformation characteristics of the flexible SAW device. **a** Resonant frequency responses of the flexible SAW device to the applied strains from 0 to 1322 με in both loading and unloading conditions, and the inset shows an excellent linearity and small hysteresis of the frequency responses; **b** resonant frequency shifts as a function of strain for flexible SAWs at different IDT wavelengths; **c** comparisons between the experimental strain sensitivities and theoretically calculated strain sensitivities (induced by velocity change, IDT deformation and their combined effect) at different IDT periods; **d** resonant frequency shifts as a function of strain for the flexible SAW device at different off-axis angles α; **e** comparison between the experimental strain sensitivities and theoretical calculated strain sensitivities as a function of the off-axis angle α; **f**, **g** comparisons between the experimental frequency shifts and theoretical calculated frequency-strain responses (induced by the initial stress, elastic constants, density and IDT deformation), at off-axis angles of α = 0° and α = 90°, respectively, for the AlN/flexible glass SAW device with *λ* = 20 μm; and (**h**) frequency responses of the fabricated flexible SAW devices during the cyclic bending test.

The electromechanical responses of the flexible SAW device (with a wavelength of 20 μm) as a function of applied strain from zero to 1332 με and then to its full recovery state are shown in Fig. 3a. The results show that the frequency change of the flexible SAW device is linearly correlated with the applied strain. The calculated linear regression coefficient is ∼0.99967, showing excellent linearity, based on which the strain sensitivity was calculated to be 99.5 Hz/με. For frequency-strain recovery in one cycle, the maximum hysteresis of the flexible SAW device is less than 0.24%, which is much better than the reported value for flexible SAW devices^11^.

To investigate the effects of the wavelength on the frequency-strain responses, flexible SAW devices with different wavelengths have been applied with different bending strains, and the obtained frequency results are shown in Fig. 3b. The corresponding strain sensitivity data were calculated to be 180.08, 130.29, 99.50, and 64.38 Hz/με for devices with wavelengths of 12, 16, 20, and 24 μm, respectively. This indicates that the strain sensitivity of AlN/glass-based flexible SAW devices decreases significantly with increasing wavelength, mainly due to the decreased resonant frequency. We have also theoretically analyzed the effects of the SAW wavelength on the strain-frequency responses, and the results are shown in Fig. S3(a). The calculated strain sensitivities are in good agreement with the experimental results, as illustrated in Fig. 3c.

Figure 3d shows the frequency shifts of the flexible AlN SAW devices as a function of bending strain, with different off-axis angles of 0, 15°, 30°, 45°, 60°, 75°, and 90°. It is obvious that the frequency-strain responses of flexible SAW devices still exhibit good linearity at different off-axis angles, and the obtained frequency-strain sensitivity decreases with increasing off-axis angle α. The theoretical results of frequency shifts as functions of bending strain and off-axis angle α are shown in Fig. S3(b), which are consistent with the experimental data, as illustrated in Fig. 3e. An interesting phenomenon is that when the off-axis angle α is 90°, the frequency variation is different from those with the other off-axis angles. A previous study on ZnO/glass SAW devices also reported this phenomenon, which was attributed to experimental error^26^. However, our flexible SAW devices with different wavelengths present the same trend, indicating that it should be a real phenomenon.

To understand the mechanisms behind this phenomenon, we theoretically calculated the frequency-strain responses of the AlN/glass flexible device (*λ* = 20 μm) by considering the initial stress, elastic constant and density, which are three key factors leading to the apparent changes in the SAW velocity. We then calculated the frequency-strain responses due to the IDT deformation (e.g., changes in IDT dimensions) in the direction of SAW propagation and the combined effects due to all the above factors. The results are shown in Fig. 3f and g. When the off-axis angle α is 0°, the IDT deformation causes a negative frequency shift, but the combined effect of IDT deformation and change in SAW velocity leads to a positive frequency shift. When the off-axis angle α is 90°, even though the IDT deformation causes a positive frequency shift, the combined effect leads to a negative frequency shift. These results clearly demonstrate that the frequency shift is mainly due to the changes in SAW velocity but not IDT deformation. In addition, both the initial stress and change in density will lead to a positive frequency shift, while the changes in elastic constants cause a negative frequency shift for the off-axis angle α between 0 and 90°.

Clearly, the changes in acoustic velocity depend on the combined effect of all three factors (e.g., initial stress, density, and elastic constants) and could be positive or negative. When the off-axis angle α is 0°, the main contribution factor on the frequency-strain shift is the initial stress (Fig. 3f). However, when the off-axis angle α is increased from 0° to 90°, the contribution of the initial stress to the positive frequency-strain shift is remarkably decreased, while the effects of the elastic constants and density on the frequency-strain shifts are only slightly changed. Therefore, the combined effect will lead to a frequency change from a positive value to a negative value when the off-axis angle α is increased from 0° to 90°.

Figure S4(a) and (b) shows the variations in insertion losses of the flexible SAW device as functions of bending strain and off-axis angle α at different wavelengths. The insertion loss of flexible SAW devices has not shown obvious changes during the bending process, showing the good stability of AlN/flexible glass-based SAW devices during bending and off-axis deformation.

To investigate the long-term stability and fatigue failure of flexible SAW devices in real-life applications, they were repeatedly bent/released for more than 1300 cycles with a maximum applied strain of 1000 με. Figure 3h shows the obtained frequency shifts at three stages: beginning stage (0–1000 s), after 9 h (36,400–37,400 s), and near the end (64,800–65,800 s) under the cyclic bending test. The performance of the SAW device (e.g., frequency shifts) does not shown apparent changes. These results verify that the fabricated flexible SAW device has good mechanical durability and stability, demonstrating its great potential for flexible electronics applications.

### Human motion detection and UV sensing on the curved surface

**Fig. 4:**
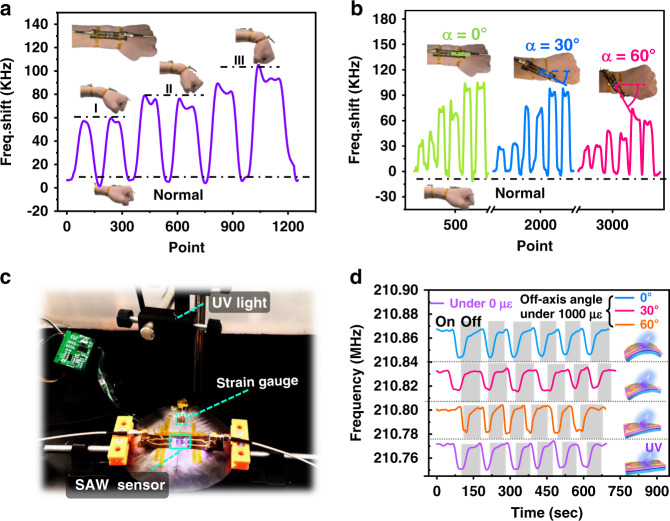
Human motion detection and UV sensing on the curved surface. **a** Frequency shifts of the flexible SAW devices at different wrist bending states; **b** frequency shifts of the flexible SAW devices at three wrist bending states at off-axis angles α of 0, 30, and 60°; **c** illustration of the experimental setup for ultraviolet detection on a curved surface using the flexible SAW sensor; and **d** frequency responses of our flexible SAW device to UV light (UV wavelength of 365 nm and intensity of 35 mw/cm²) under strains of 0 με and 1000 με, with off-axis angles α of 0, 30, and 60°.

To verify the proposed model of the flexible SAW device during bending and off-axis deformation and demonstrate its practical application, an AlN/glass flexible sensor was attached onto the wrist of a volunteer (with off-axes of 0°, 30°, 60°) to detect the bending of their wrist. The obtained results are shown in Fig. 4a. The results show that the frequency shifts of the flexible SAW device increase with increasing bending angle of the wrist, mainly due to increases in the strains. Furthermore, as the off-axis angle (α) increased from 0°, 30°, to 60°, the frequency shifts were reduced at the given bending strain. This is mainly due to the lower frequency-strain sensitivity at a higher off-axis angle, which agrees well with the strain analysis results shown above.

To demonstrate the sensing performance of flexible SAW devices that are applied onto curved and complex surfaces, the AlN/glass device, which was precoated with ZnO nanowires (purchased from XFNANO, Nanjing, China) onto the AlN film surface to enhance the absorption of UV light, was bonded onto a flat steel plate. The steel plate was in a flat state or was bent with a strain of 1000 με with off-axis angles of 0°, 30°, and 60°. Detection of the UV source (with a wavelength of 365 nm and intensity of 35 mw/cm²) was performed using the above device at different bending conditions, and the obtained results are shown in Fig. 4c. The results show that the off-axis angle α and bending strain will affect the starting/resonant frequency of the flexible SAW device, but the UV performance is almost the same and does not have obvious differences for all the cycling tests. Our results demonstrated that the fabricated SAW device can work well at different off-axis angles α and bending strains and has shown a comparable UV sensing performance compared with those on the flat plate surface.

It is worthwhile to note that the frequency shifts are related to both the bending strain and off-axis angle. To distinguish the individual effect of these two parameters on the frequency responses, we can use a pair of identical SAW devices, which are orthogonally positioned in the sensing area. After applying a bending strain and an off-axis deformation to the area, these two orthogonal SAW devices will produce different frequency shift results. We can solve/decouple from the individual frequency shifts of these two orthogonal SAW devices and identify the effects of the bending strain and off-axis deformation.

## Conclusion

In this work, we developed AlN/glass flexible SAW devices by using high-quality AlN films on flexible glass substrates. A theoretical model was first developed using the coupling wave equation and boundary condition method to systematically analyze the bending and off-axis deformation characteristics of flexible SAW devices under elastic strain. The functions of the frequency shifts in regard to different bending strains and off-axis angles were obtained, and the results were in good agreement with those from the theoretical calculations. We performed proof-of-concept demonstrations by detecting human wrist bending at various off-axis angles and by sensing UV light changes on a curved surface, thereby proving the great potential of the device for application in versatile and flexible electronics.

## Supplementary information

Supplemental Material
