# Supplementary material for: Flexible thin-film acoustic wave devices with off-axis bending characteristics for multisensing applications
Source: Microsyst Nanoeng. 2021 Nov 26;7:97. doi: 10.1038/s41378-021-00325-3 (PMC8626450; doi:10.1038/s41378-021-00325-3)
Supplement: Supplementary file 1 — Supplemental Material [file 41378_2021_325_MOESM1_ESM.docx]

Supporting Information

**Thin Film Flexible Acoustic Wave Devices with Off-axis Bending Characteristics for Multi-Sensing Applications**

*Zhangbin Ji*^1^, *Jian Zhou*^1,*^, *Huamao Lin^2^*, *Jianhui Wu*^1^, *Dinghong Zhang^1^, Sean Garner^3^,* *Alex Gu^2^,Shurong Dong^4^, Yongqing Fu*^5^*,* *Huigao Duan*^1,*^

^1^ College of Mechanical and Vehicle Engineering, Hunan University, Changsha 410082, China

^2^ Shanghai Industrial μTechnology Research Institute (SITRI), 235 Chengbei Rd, Shanghai, 201800, China

^3^Corning Research & Development Corporation, One River Front Plaza, NY 14831, USA

^4^College of Information Science and Electronic Engineering, Zhejiang University, Hangzhou 310027, China

^5^Faculty of Engineering and Environment, Northumbria University, Newcastle upon Tyne, NE1 8ST, United Kingdom

*E-mail: [jianzhou@hnu.edu.cn](mailto:jianzhou@hnu.edu.cn), [duanhg@hnu.edu.cn](mailto:duanhg@hnu.edu.cn)

This file includes:

1. Boundary conditions and bending perturbation analysis of flexible SAW devices

2. Frequency responses of flexible SAW devices with different wavelengths

**3. Performance comparisons of AlN/glass SAW devices with those based on ZnO films, Al foils, and flexible polymers.**

4. Calculated results of frequency shifts of flexible SAW devices as a function of bending strain at different wavelengths and off-axis angles

5. Variations of insertion losses of flexible SAW devices as a function of bending strain at different wavelengths and off-axis angles

**6. Comparisons of performance parameters from our flexible sensors with those from other human wrist movement sensors and UV sensors**

1. **Boundary conditions and bending perturbation analysis of flexible SAW devices**

AlN/flexible glass based flexible SAW device satisfies the following boundary conditions:

$u_{k}\left( L \right)|_{x_{3}\left( L \right)=-h}= u_{k}\left( S \right) |_{x_{3}\left( s \right)=0}$

$T_{3j}^{'}\left( L \right)|_{x_{3}\left( L \right)=-h}=T_{3j}^{'}\left( s \right)|_{x_{3}\left( s \right)=0}$

$$T_{3j}^{'}\left( L \right)|_{x_{3}\left( L \right)=0}=0$$

$D_{3}\left( |_{x_{3}\left( L \right)=0} \right)|_{layer}=D_{3}\left( |_{x_{3}\left( L \right)=0} \right)|_{vacuum}$ (S1)

1. The continuity of electric potential $\varphi$ and electric displacement $\boldsymbol{D}_{\boldsymbol{3}}$ at $x_{3}(L)$=0 and $\varphi$vanishes at $x_{3}(L)$=H.

$\sum_{q=1}^{Q\left( L \right)} B_{\left( S \right)}^{\left( q \right)}[\sum_{l=1}^{3} \sum_{k=1}^{4} \text{M}_{\text{3jkl}}\left( L \right)\gamma_{l\left( L \right)}^{\left( q \right)}\beta_{k\left( L \right)}^{\left( q \right)}+j\varepsilon_{0}\beta_{4\left( L \right)}^{\left( q \right)}\coth\left( \frac{\omega H}{v} \right){]B}_{(L)}^{(q)}\text{=0 }$ (S2)

1. Continuity of particle displacement and electric potential at the interface between the thin piezoelectric layer and the substrate follows the following relationship.

$\sum_{q=1}^{Q\left( L \right)} e^{j\omega t}B_{\left( L \right)}^{\left( q \right)}\beta_{k\left( L \right)}^{\left( q \right)}exp\left[ j\frac{w}{v}\left( \gamma^{\left( q \right)}h \right) \right]-\sum_{q=1}^{Q\left( S \right)} B_{\left( S \right)}^{\left( q \right)}\beta_{k\left( S \right)}^{\left( q \right)}=0$ (k=1-4) (S3)

1. Continuity of normal stress and electric displacement components at the interface between the thin piezoelectric layer and the substrate follows the following relationship.

$\sum_{q=1}^{Q\left( L \right)} B_{\left( L \right)}^{\left( q \right)}\sum_{l=1}^{3} [\sum_{k=1}^{4} \text{M}_{\text{3jkl}}\left( L \right)\gamma_{l\left( L \right)}^{\left( q \right)}\beta_{k\left( L \right)}^{\left( q \right)}+\gamma_{l}^{\left( q \right)}\sigma_{3l}(1-\delta_{j4}){.\beta}_{j(L)}^{(q)}].\text{exp(}\frac{\mathrm{jwh}}{v}\gamma_{3(L)}^{(q)}\text{) }-\sum_{q=1}^{Q\left( S \right)} B_{\left( S \right)}^{\left( q \right)}\sum_{l=1}^{3} [\sum_{k=1}^{4} \text{M}_{\text{3jkl}}\left( S \right)\gamma_{l\left( s \right)}^{\left( q \right)}\beta_{k\left( S \right)}^{\left( q \right)}+\gamma_{l}^{(q)}\sigma_{3l}(1-\delta_{j4}){.\beta}_{j(s)}^{(q)}]\text{=0}$ (j=1-4) (S4)

1. Zero traction force at the free surface of the piezoelectric layer can be described using.

$\sum_{q=1}^{Q\left( L \right)} B_{\left( S \right)}^{\left( q \right)}\sum_{l=1}^{3} [\sum_{k=1}^{4} M_{3jkl}\left( L \right)\gamma_{l\left( S \right)}^{\left( q \right)}\beta_{k\left( s \right)}^{\left( q \right)}+\gamma_{l}^{(q)}\sigma_{3l}(1-\delta_{j4}){.\beta}_{j(S)}^{(q)}]=0$ (j=1-3) (S5)

For bending perturbation analysis of the flexible SAW devices, the applied strain is a perturbation to the propagating SAWs, thus inducing the changes in the SAW velocity (v). Three independent factors may contribute to the changes of the SAW velocity, including the initial stress ($\sigma_{\mathrm{jk}}$) described by the modified motion equation, elastic constant ($C_{\mathrm{ijkl}}^{E}$) change of AlN/glass, and the density (ρ) change of AlN/glass. According to the elastic perturbation theory, the perturbed elastic constants are determined by equation $C_{ij}^{'}=C_{ij}$+$C_{ijk}\cdot\varepsilon_{k}$ (i, j, k = 1-6), where $C_{ijk}$are the third order elastic constants. $\varepsilon_{k}$is the Euler strains. The perturbed density can be descried by equation $\boldsymbol{\rho}^{\boldsymbol{'}}$= $\rho-d\rho=$ $\rho-\frac{m}{x^{2}yz}dx-\frac{m}{xy^{2}z}dy-\frac{m}{xyz^{2}}dz$.=$\rho-\rho\cdot（\varepsilon_{1}+\varepsilon_{2}+\varepsilon_{3}）$where $\varepsilon_{1}{,\varepsilon}_{2} and \varepsilon_{3}$ are the strain components in three dimensions.

Table S1 Material parameters of AlN thin film and flexible glass

| **Material parameters of AlN thin film and flexible glass** | | |
| --- | --- | --- |
|  | **AlN thin film** | **Flexible glass** |
| Density(kg/m³) | **3260** | **2380** |
| Elastic stiffness  Constants C (Gpa) | $\boldsymbol{C}_{\boldsymbol{11}}$**=345** $\boldsymbol{C}_{\boldsymbol{12}}$**=125** $\boldsymbol{C}_{\boldsymbol{13}}$**=120**  $\boldsymbol{C}_{\boldsymbol{33}}$**=395** $\boldsymbol{C}_{\boldsymbol{44}}$**=118** $\boldsymbol{C}_{\boldsymbol{66}}$**=110** | $\boldsymbol{C}_{\boldsymbol{11}}$**=85** $\boldsymbol{C}_{\boldsymbol{12}}$**=25.4** |
| dielectric constants （F/m） | $\boldsymbol{\varepsilon}_{\mathbf{11}}\mathbf{=8.0}\boldsymbol{\varepsilon}_{\mathbf{33}}\mathbf{=9.0}$ | **5.5** |
| Piezoelectric Constants  (N/m^2^) | $\text{e}_{\text{15=}}\text{-0.48 }\text{ e}_{\text{31=}}\text{-0.48}$  $\text{e}_{\text{33=}}\text{-0.48}$ | **0** |

2. Frequency responses of flexible SAW Devices with different wavelengths

Figure S1. Transmission spectra of the flexible AlN/glass based SAW devices as a function of wavelength λ: (a) λ =12 μm; (b) 16 μm; (c) 20 μm; and (d) 24μm

**3. Performance comparisons of AlN/glass SAW devices with those based on ZnO films, Al foils, and flexible polymers**

The performance of the AlN/glass SAW device is determined by several parameters, such as the film substrate materials, film quality, film thickness, frequency and SAW electrode design for different applications. For ZnO film on flexible polymers such as polyimide (PI) and polyethylene terephthalate (PET), the thickness of the ZnO film needs to be larger than ~1.2 μm, and the larger thickness lead to a better device performance^1^, as shown in Figure S2(a). In this paper, our AlN film is only 1.6 μm, compared with the previously reported ZnO (1.7 μm, see Fig. S2(a)) based flexible SAW on PI, our AlN/flexible glass SAW has a better performance, due to the significant dissipation of sound waves and energy into the polymer substrate. We further conducted experiments using 2.3 μm AlN/ glass SAW devices, and the results are shown in Figure S2(b), which reveals a value of 28 dB for the signal amplitude of S_21_ (out of band rejection). It is clear that compared with that of the 2.3 μm ZnO/glass SAW device (with only 10 dB for signal amplitude of S_21_), our 2.3 μm AlN film based SAW device has shown a much better performance. We also compared our AlN/flexible glass SAW devices with other reported results for 5 μm ZnO/Al foil based SAWs (Figure S2(c), Ref.2) and 4.5 μm AlN/PEN based SAWs (see Figure S2(d), Ref.3), and the results show that our AlN/flexible glass based SAWs have a much larger signal amplitude of S_21_ (out of band rejection), indicating a better performance.

Figure S2. (a) Effects of ZnO film thickness on the resonant spectra of the ZnO/PI based flexible SAW devices^1^ (Copyright 2013, American Institute of Physics), showing that the larger the film thickness, the better the device’s performance; (b) Transmission spectrum of our fabricated AlN/flexible glass based SAW device with AlN film of 2.3 μm thick; (c) Transmission spectra of the flexible acoustic wave devices with a ZnO/foil structure^2^(Copyright 2015, Elsevier Science Publisher B.V.); (d) Transmission spectra of the flexible acoustic wave devices with an AlN/PEN structure^3^ (Copyright 2016, Springer New York LLC).

4. Calculated results of frequency shifts of flexible SAWs as a function of bending strain at different wavelengths and off-axis angles

Figure S3. The calculated results of frequency shifts of flexible SAW devices: (a) as a function of bending strain at different wavelengths with off-axis angle α of 0°; (b) as a function of bending strain at different off-axis angles α with a wavelength of 20 μm

5. Variations of insertion loss of flexible SAW as a function of bending strain at different wavelengths and off-axis angles

Figure S4. (a) Variation of insertion loss of flexible SAW devices as a function of bending strain at different wavelengths； (b) Variation of insertion loss of flexible SAW device as a function of bending strain at different off-axis angles

**6. Comparisons of performance parameters of our flexible sensors with those of other human wrist movement sensors and UV sensors**

Many researchers have been focusing on the research of human wrist movement sensors. However, currently, few SAW based strain sensors have been applied for human wrist movements. The main reason is because that the conventional SAW device is rigid, and not flexible. Compared our flexible SAW sensors with other type of human wrist movement sensor, such as flexible resistance type sensor and flexible capacitive type sensor, our SAW devices have both advantages and disadvantages, which has been listed in Table SⅡ.

It is quite hard to compare the sensitivities of different types of sensors, as our sensor detects the frequency shifts, whereas the others detect the changes of resistance or capacitive values. For the linearity and hysteresis, our device’s sensing linearity is about 99.8%, and the hysteresis is about 0.24%, both of which are much better than many other types of human wrist movement sensors^4-11^. In addition, the SAW sensor is a unique MEMS sensor with the advantage of wireless and passive detection. Moreover, the ultra-thin flexible glass based SAW device do not have any soft polymer materials, and have the potentials for wafer-level production (up to 6-inch scale).

However, it should be addressed that the SAW sensors need a relatively more complex signal acquisition circuit compared with those of the other kinds of sensors such as resistance type sensors. Furthermore, our SAW sensor cannot be stretched significantly, whereas many other human wrist movement sensors can be stretched easily.

**Table SⅡ.** Comparisons of Device Performance of Differtent Strain sensors

| **Sensor type** | **Sensitiviy** | **Hysteresis** | **Linearity(R^2^)** | **Ref.** |
| --- | --- | --- | --- | --- |
| Resistive | GF=5 | >20% | 94% | 4 |
| Resistive | GF=2~4 | 0.15% | 99.90% | 5 |
| Capacitive | GF=-2 | >15% | 99.68% | 6 |
| Resistive | GF=47 | / | 99.47% | 7 |
| Resistive | GF=8767.4 | >4% | 97.1% | 8 |
| Capacitive | GF=0.969 | >2% | / | 9 |
| Resistive | GF=870 | >2% | 99.6% | 10 |
| Resistive | GF=1148 | >4% | 98.5% | 11 |
| Flexible SAW | 180.08με/Hz | 0.24% | 99.99% | **This work** |

On the other hand, for the UV detection, there are indeed many previous studies ^12-17^, as listed in Table SⅢ. It is clear that our flexible SAW UV sensor has not shown the best performance. However, most of previous SAW UV sensors are rigid. Unlike our flexible devices, they cannot be bent, and cannot be attached to the curved surface for UV monitoring.

**Table SⅢ.** Comparisons of UV light detectors based on SAW devices.

| **substrate type** | **Resonance mode** | **Resonance**  **frequency (MHz)** | **Sensitivity**  **(mw/cm^2^)^−1^** | **Ref.** |
| --- | --- | --- | --- | --- |
| Al foil | Rayleigh | 78.48 | 3.89ppm | 12 |
| LiNbO_3_ | Rayleigh | 37 | 40ppm | 13 |
| Quartz | Rayleigh | 41.2 | 19ppm | 14 |
| Si | Rayleigh | 122.15 | 3ppm | 15 |
| Si | Rayleigh | 180.71 | 0.6ppm | 16 |
| flexible glass | Rayleigh | 169.44 | 1.66ppm | 17 |
| **flexible glass** | **Rayleigh** | **211.77** | **2.8ppm** | **This work** |

# REFERENCES

1. Zhou, J., et al., Crystalline structure effect on the performance of flexible ZnO/polyimide surface acoustic wave devices. *J. Appl. Phys.* **114**, 044502 (2013).

2. Liu, Y., et al., Flexible and bendable acoustofluidics based on ZnO film coated aluminium foil. *Sens. Actuators, B*, **221**, 230-235 (2015).

3. Li, Q., et al., Growth and Characterization of Polyimide-Supported AlN Films for Flexible Surface Acoustic Wave Devices. *J. Electron. Mater.* **45**, 702-2709 (2016).

4. Amjadi, M., et al., Highly Stretchable and Sensitive Strain Sensor Based on Silver Nanowire-Elastomer Nanocomposite. *ACS Nano*, **8**, 5154-5163. (2014).

5. Choi, D.Y., et al., Highly Stretchable, Hysteresis-Free Ionic Liquid-Based Strain Sensor for Precise Human Motion Monitoring. *ACS Appl. Mater. Interfaces,* **9**, 770-1780 (2017).

6. Kim, S.R., J.H. Kim, and J.W. Park, Wearable and Transparent Capacitive Strain Sensor with High Sensitivity Based on Patterned Ag Nanowire Networks. *ACS Appl. Mater. Interfaces,* **9**, 26407-26416 (2017).

7. Liu, X., et al., Highly Sensitive and Stretchable Strain Sensor Based on a Synergistic Hybrid Conductive Network. *ACS Appl. Mater. Interfaces,* **12** 42420-42429 (2020).

8. Shi, X., et al., Bioinspired Ultrasensitive and Stretchable MXene-Based Strain Sensor via Nacre-Mimetic Microscale "Brick-and-Mortar" Architecture. *ACS Nano*, **13**, 649-659 ( 2019) .

9. Park, S., et al., Stretchable energy-harvesting tactile electronic skin capable of differentiating multiple mechanical stimuli modes. *Adv. Mater.* **26**, 7324-32 (2014).

10. Wang, Y.F., et al., Printed Strain Sensor with High Sensitivity and Wide Working Range Using a Novel Brittle-Stretchable Conductive Network. *ACS Appl Mater Interfaces*, **12**, 35282-35290 (2020).

11. Yang, Y., et al., Ti3C2Tx MXene-graphene composite films for wearable strain sensors featured with high sensitivity and large range of linear response. *Nano Energy,* **66**, 104134 (2019).

12. Tao, X., et al., Three-Dimensional Tetrapodal ZnO Microstructured Network Based Flexible Surface Acoustic Wave Device for Ultraviolet and Respiration Monitoring Applications. *ACS Appl. Nano Mater*. **3**, 1468-1478 (2020).

13. Wei, C.-L., et al., UV detection based on a ZnO/LiNbO3 layered surface acoustic wave oscillator circuit. *J. Vac. Sci. Technol., A*. **27**, 1343-1346 (2009).

14. Kumar, S., et al., ZnO based surface acoustic wave ultraviolet photo sensor. *J. Electroceram.* **22**, 198-202 (2008).

15. Phan, D.-T. and G.-S. Chung, Characteristics of SAW UV sensors based on a ZnO/Si structure using third harmonic mode. *Curr. Appl. Phys*. **12**, 210-213 (2012).

16. Guo, Y.J., et al., Ultraviolet sensing based on nanostructured ZnO/Si surface acoustic wave devices. *Smart Mater. Struct.* **24**, 125015 (2015).

17. Yin, C.S., et al., Enhancing the sensitivity of flexible acoustic wave ultraviolet photodetector with graphene-quantum-dots decorated ZnO nanowires. *Sens. Actuators, A.* **321**, 112590 (2021).
